# M2 Genotype of *Varicella zoster* virus associated with Severe Herpes zoster in Pakistan

**DOI:** 10.12669/pjms.40.6.8764

**Published:** 2024-07

**Authors:** Saba Hanif, Ayesha Isani Majeed, Rani Faryal

**Affiliations:** 1 Ms. Saba Hanif, Department of Microbiology, Faculty of Biological Sciences, Quaid-i-Azam University, Islamabad, Pakistan; 2 Prof. Dr. Ayesha Isani Majeed, MBBS, MCPS, FRCR, MPH, MHPE, Ph.D. Department of Radiology and Breast Cancer Screening Programme, Pakistan Institute of Medical Science, Islamabad, Pakistan; 3 Prof. Dr. Rani Faryal, Ph.D., Postdoc Department of Microbiology, Faculty of Biological Sciences, Quaid-i-Azam University, Islamabad, Pakistan

**Keywords:** herpes zoster, M2-genotype, acyclovir, renal failure, Pakistan

## Abstract

This case report is of herpes zoster which is caused by Varicella zoster virus (VZV). The patient was presented with acute renal failure associated with intravenous acyclovir administration for its management. A 50 years old man visited the hospital with rashes on his back. The serum sample was positive for anti-VZV IgM via Enzyme Linked Immunosorbent Assay (ELISA), and vesicular swab for VZV via polymerase chain reaction (PCR). Phylogenetic analysis identified it as M2-genotype. Patient was treated with intravenous acyclovir administration, which led to acute renal failure. Later with shift to oral acyclovir, renal functions were restored. Elderly patients with reactivation of VZV in Pakistan are at risk to contract herpes zoster. Acyclovir is drug of choice via intravenous route was found to be nephrotoxic, however oral acyclovir was safe and effective. This is first report on pathogenic VZV genotype from Pakistan and is presented to highlight that the herpes zoster cases of elderly patients’ treatment option need to be revisited.

## INTRODUCTION

Herpes zoster or shingles is one of the most contagious diseases presented as painful, fluid filled maculo-papular rash, caused by reactivation of *Varicella zoster* virus, which remains dormant in cranial and spinal ganglia during primary infection. According to an estimate, more than 10% of shingles patients experience complications like post-herpetic neuralgia, cerebro-vascular complications and organ failure.^1^

Acyclovir drug is primarily recommended to treat herpes zoster infection, which can cause nephrotoxicity specifically a reversible renal failure.^2^ The incidence of acute kidney injury associated with intravenous drug administration is found to be 12-48% of patients.^3^ Case reports for acyclovir treatment followed by renal failure have been reported from different countries. A case study of 35 years-old woman with herpes zoster from China, developed kidney failure after valacyclovir administration.^4^ To attain successful management of disease, accurate investigation, molecular identification of underlying causative agent, and proper treatment is critical. There is lack of information available to health care providers on risk and treatment strategies among elderly population. Studies have reported VZV in elderly population individuals; however, data on detection and genotypes is still lacking from Pakistan. Therefore, the purpose of this case report was to highlight the significance of VZV genotyping, and adverse side effects of antivirals along with associated herpes zoster complications.

## CASE REPORT

A 50 years old man from Gujrat (Pakistan), with herpes zoster, who had developed acute renal failure after intravenous acyclovir treatment is presented in this study. The study was approved by ethical review board of the Quaid-i-Azam University, Islamabad (No.BEC-FBS-QAU-2017). Written consent was obtained from the patient for clinical evaluation. Demographic, clinical and laboratory data was obtained to maintain the medical record, also blood sample and vesicular swab was collected for the laboratory investigation.

**Fig.1:**
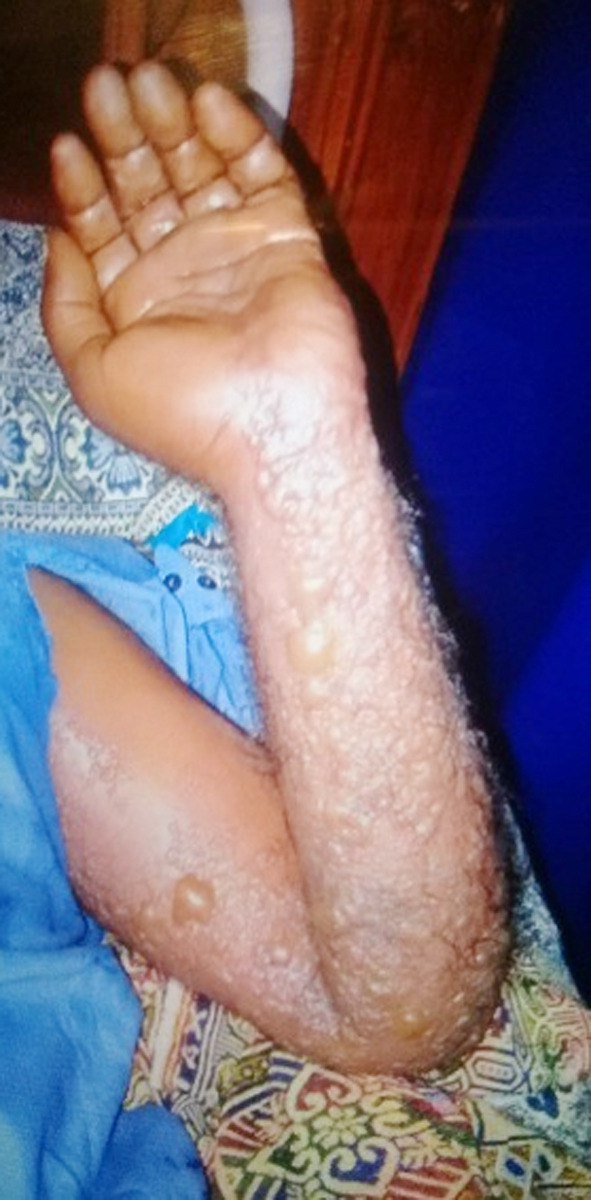
Maculopapular raised, fluid filled vesicular lesions at multiple dermatomes of left arm of a 50-years old male patient clinically suspected with herpes zoster.

The patient visited Dermatology Outpatient Department of Pakistan Institute of Medical Sciences, Islamabad, with complaint of rash on left side of his back. After initial examination, he was admitted to the isolation Medical ward of Infectious Disease Department at the hospital because of suspected viral infection. His medical history showed no previous shingles vaccination and was immunocompetent. The patient developed symptoms two to three days before the hospital visit. After two days of hospital admission, he developed fluid filled lesions on his left arm too (Fig.1).

Patient’s blood sample was sent to Department of Virology, National Institute of Health (Islamabad). Upon serological analysis, the serum sample found negative for Measles, Rubella and Dengue; however, was detected positive for anti-VZV IgM using the ELISA kit (Vircell Spain, Cat#19EVZV101). To confirm the causative pathogen, vesicular lesion swab was analyzed at Molecular Medicine Laboratory (Department of Microbiology, Quaid-i-Azam University, and Islamabad) after DNA extraction via QIAmp DNA Mini Kit (Cat#51306). The PCR followed by Sanger sequencing was carried out. Amplification of targeted regions of three ORFs; ORF 22 (37837-38356), ORF21 (33497-33999) and ORF 54 (95109-95330) was performed to identify the causative agent and its genotype. Amplified and sequenced DNA was analyzed by pairwise sequence alignment by using Bio-Edit v-4. Based on single nucleotide polymorphisms in ORF21 (33722; C, 33725; C, 33728; C), ORF22 (38055; C, 38081; C, 38177; A) and ORF54 (95241; C), M2-genotype of VZV was confirmed to be associated with the said condition. The sequences generated were submitted to NCBI GeneBank database under the accession number OP946971, PP295254, and PP295253 for ORF22, ORF21 and ORF54 respectively.

**Fig.2:**
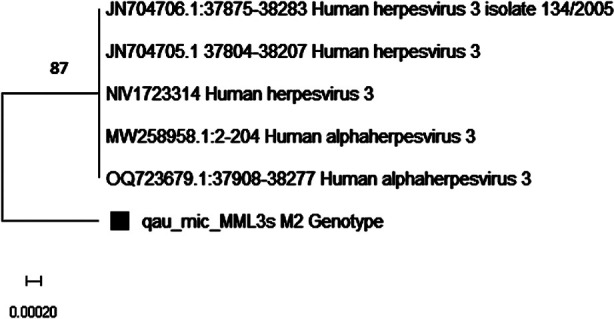
Phylogenetic analysis of VZV strain isolated from herpes zoster patient of the study by Neighbor Joining Method with 500 replications with Tamura Nei Model. Number in the tree represented the bootstrap value. The evolutionary distances were computed using the p-distance method.

A phylogenetic tree was constructed based on the nucleotides of the ORF22 (37837-38356) region using MEGA-X to depict the evolutionary relationship of the VZV genotype by comparing it with sequences obtained from the Gene Bank database. The phylogenetic analysis showed 99% homology of this strain (qau_mic_MML3s) with the M-genotype VZV strains from India (NIV1723314, OQ723679), one of the neighboring country. The homology of 93% and 95% was seen with the M-genotype VZV strains from Germany (JN704705, JN704706) and Belarus (MW258958) respectively. The bootstrap values are given in Fig.2.

Meanwhile, treatment was started with antivirals (acyclovir injection-500mg), antibiotics (Tanzo-4.5g), anti-depressor (tryptanol-25mg), polyfex plus and gabapentin-100mg. Painful, red and raised vesicular lesions were spread to multiple dermatomes of left arm after 14days. Following third week small lesions remained intact, whereas, large lesions ruptured following bleeding. After 15 days, he was prescribed with acyclovir tab 800mg for one week.

Serum analysis during the intravenous administration of acyclovir showed elevated level of creatinine 8.9mg/dL. His clinical condition deteriorated with development of acute renal failure as his creatinine level was kept on increasing with intravenous drug administration and had to undergo dialysis twice a week. However, it was reduced to 5.3mg/dL within three to four days after switching to oral acyclovir and reached a normal value after a week of oral acyclovir administration. Patient’s condition progressively improved following third week of admission. After a month, he was discharged as his ruptured skin started healing, and kidneys had regained normal function.

## DISCUSSION

We have presented a case report of suspected herpes zoster in a 50 years old patient with acute renal failure upon intravenous acyclovir administration. Patient recovered and restored normal renal function after a month and was discharged from hospital. Previously, no genotyping of circulating VZV strains was done in Pakistan. However, studies from South Asia reported M genotype as the most prevalent circulating genotype, and the J genotype being the most prevalent from East Asia.^5^ Only live attenuated vaccine based on J genotype is available and effective in preventing both varicella and herpes infection by all genotypes.^6^ Our study confirmed the presence of M2-genotype in a herpes zoster patient. Based on this detected M2-genotype and associated clinical presentation, an effective treatment strategy can be implemented along with the administration of vaccine and proper dosage of antivirals in elderly people.

To the of knowledge of authors, although data is available in literature regarding herpes zoster followed by renal failure upon acyclovir administration, but no such data was reported from Pakistan. This is the first case report from Pakistan with acute renal failure after intravenous acyclovir drug administration for herpes zoster treatment and needed weekly dialysis. This might be due to drug crystallization and precipitation in renal tubules leading to tubular obstruction.^7^ Similarly, two case studies reported; one from Japan and other from Africa, where elderly patients in eighties developed acute kidney injury with herpes zoster infection, when treated with valacyclovir.^8^,^9^

Our patient also suffered with impaired renal function, when acyclovir was administered intravenously, and later the normal kidney function was restored, when shifted to oral drug. Similarly a population based study from Canada found no risk of renal failure with oral acyclovir administration.^10^ The effective dosage of oral or intravenous acyclovir drug administration is still questionable and detailed focused studies based on relationship between the VZV genotype and clinical presentation of disease are needed for better understanding in herpes zoster cases.

## CONCLUSION

In summary**,** this is the first report on VZV genotype responsible for severe herpes zoster infection from Pakistan. This also gives insight on treatment failure with intravenous acyclovir leading to acute renal failure in older persons. This case study tries to bridge the gap on understanding effects of prescribed antiviral in local hospital settings.

### Recommendations:

It is suggested that the health authorities must establish active surveillance system coupled with well-established laboratory investigation, vaccination and alternative treatment strategies to control severe herpes zoster infection and its outcomes in Pakistani population.

### Authors` Contribution:

**SH** conceived, designed & editing of manuscript, is responsible for integrity of research

**AIM** did data collection and manuscript writing

**RF** designed the research work, review, approved and responsible for integrity of research.
